# The Efficacy of eHealth Interventions for the Treatment of Adults Diagnosed With Full or Subthreshold Binge Eating Disorder: Systematic Review and Meta-analysis

**DOI:** 10.2196/17874

**Published:** 2021-07-20

**Authors:** Elnaz Moghimi, Caroline Davis, Michael Rotondi

**Affiliations:** 1 School of Kinesiology and Health Science Faculty of Health York University Toronto, ON Canada

**Keywords:** internet, cognitive behavioral therapy, guided self-help, obesity, weight loss, eating disorder, binge eating, mobile phone

## Abstract

**Background:**

There has been a recent rise in the use of eHealth treatments for a variety of psychological disorders, including eating disorders.

**Objective:**

This meta-analysis of randomized controlled trials is the first to evaluate the efficacy of eHealth interventions specifically for the treatment of binge eating disorder (characterized by compulsive overconsumption of food, in a relatively short period, and without compensatory behaviors such as purging or fasting).

**Methods:**

A search on the electronic databases PubMed, Web of Science, Embase, MEDLINE, and CINAHL was conducted for randomized controlled trials that compared the efficacy of eHealth treatment interventions with waitlist controls.

**Results:**

From the databases searched, 3 studies (298 participants in total) met the inclusion criteria. All interventions were forms of internet-based guided cognitive behavioral therapy. The results of the analysis demonstrated that when compared with waitlist controls, individuals enrolled in eHealth interventions experienced a reduction in objective binge episodes (standardized mean difference [SMD] −0.77, 95% CI −1.38 to −0.16) and eating disorder psychopathology (SMD −0.71, 95% CI −1.20 to −0.22), which included shape (SMD −0.61, 95% CI −1.01 to −0.22) and weight concerns (SMD −0.91, 95% CI −1.33 to −0.48). There was no significant difference in BMI between the eHealth interventions and controls (SMD −0.01, 95% CI −0.40 to 0.39).

**Conclusions:**

These findings provide promising results for the use of internet-based cognitive behavioral therapy for binge eating disorder treatment and support the need for future research to explore the efficacy of these eHealth interventions.

## Introduction

### Background

Binge eating disorder (BED) is recognized in the Diagnostic and Statistical Manual of Mental Disorders as abnormal and excessive eating patterns marked by uncontrolled, recurrent, and persistent binge eating [1]. In the individual, binge eating evokes guilt and distress but without compensatory weight loss behaviors (eg, purging), such as those found in individuals with bulimia nervosa. It is the most commonly diagnosed eating disorder (ED) compared with anorexia and bulimia nervosa [2] and is estimated to have a global pooled prevalence of 0.9%, with prevalence rates higher in women (1.4%) than in men (0.4%) [3].

BED is of particular research interest because of its frequency in primary care, its comorbidity with obesity and other medical and psychiatric disorders, and its high socioeconomic impact as a result of reduced quality of life and an increased need for patients to use health and medical services [4-6]. Individuals diagnosed with BED have higher inpatient, hospital-based outpatient, and prescription-medication utilization and expenditure compared with age- and sex-matched controls, both before and after their BED diagnosis [7]. The health care utilization found in patients with BED is comparable with that found in people with other EDs and major psychiatric disorders [8]. Therefore, it is imperative that effective treatments are available for these patients to help reduce health care costs and provide long-term benefits.

The current *gold standard*, evidence-based treatment for BED is cognitive behavioral therapy (CBT). CBT has been shown to reduce binge eating frequency, lead to mild weight reduction [9,10], and cause complete abstinence in 50%-60% of patients post treatment [11,12]. Despite efficacious therapies available for BED, there continues to be low rates of help seeking for this debilitating mental health condition [13]. The important reasons for these trends may be the personal feelings of shame and fear, ED-related beliefs and perceptions, and a lack of access or availability of the treatment [14,15]. Thus, it is important that novel therapies address these barriers to treatment seeking.

Recently, the use of eHealth technology has been proposed as a potentially effective alternative to traditional, in-person treatment delivery for those with BED [16-19]. Although the term *eHealth* has many facets, it is generally defined as the use of emerging information and communication technology, particularly via the internet, to improve or enable health and health care [20]. Examples include, but are not limited to, email; software programs; teleconferencing or web conferencing; digital and mobile communication; and computer, mobile, or internet apps. The benefit of eHealth treatments is that they can be administered easily, are more accessible (particularly for patient groups that do not live near urban centers), can be used anonymously, and may reduce feelings of shame and fear [15]. Furthermore, eHealth treatments may be more cost-effective because in-person treatments often require expensive resources and infrastructure [15,21,22]. Although the first internet-delivered psychological treatments only emerged in the late 1990s [23], reviews exploring the effectiveness of internet-delivered treatments for EDs have yielded positive results in terms of their impact on quality of life, binge eating, compliance, dropout, and related psychopathology [24-28].

Many eHealth treatments for BED are in line with the CBT principles described in the self-help book *Overcoming binge eating* [29]. Some studies have described this approach as internet-based cognitive behavioral therapy (I-CBT), whereas other studies have described this approach as internet-based guided self-help (I-GSH). Both therapies consist of a combination of web-based psychoeducation, writing assignments or modules, and self-monitoring such as daily eating and activity diaries [10,30-34]. In most studied cases, I-CBT and I-GSH are typically guided to varying degrees by therapists, who provide support and answer questions and concerns that the patient may have.

### Objective

Although several studies have examined the effects of eHealth treatments on different elements of BED, including bulimia [10,24,30-35], the overall effectiveness of these treatments, for this unique clinical population, has yet to be explored. Therefore, the objective of this systematic review and meta-analysis is to determine the effectiveness of eHealth treatments in adults diagnosed with full or subthreshold BED. Specifically, important hallmarks of the disorder, including binge episodes, BMI, and ED psychopathology, will be explored.

## Methods

### Search Strategy

**Figure 1:**
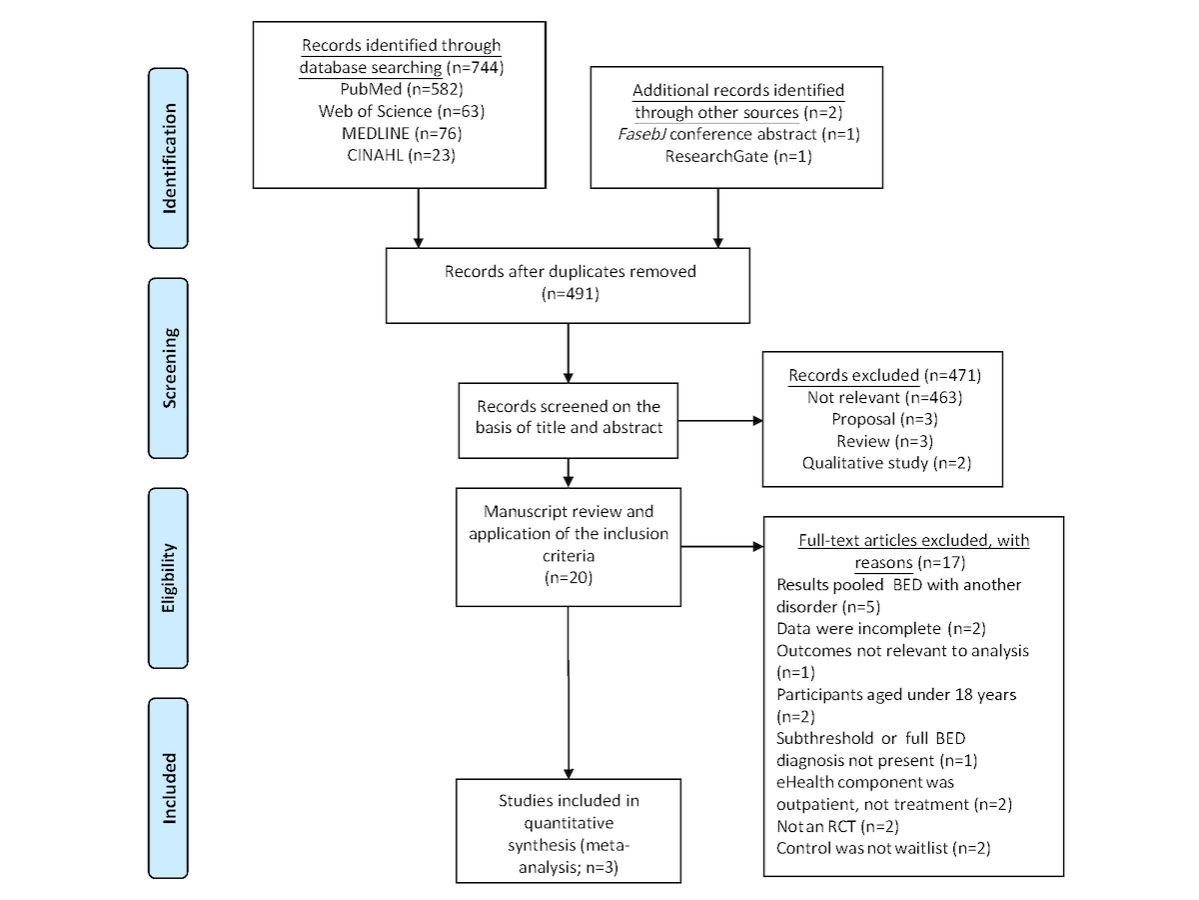
PRISMA (Preferred Reporting Items for Systematic Reviews and Meta-Analyses) flowchart outlining the selection of studies included in the meta-analysis. BED: binge eating disorder; RCT: randomized controlled trial.

PRISMA (Preferred Reporting Items for Systematic Reviews and Meta-analyses) guidelines [36] (Multimedia Appendix 1) were followed when searching the electronic databases PubMed, Web of Science, Embase, MEDLINE, and CINAHL. A combination of MeSH (Medical Subject Headings) terms and keywords that represented the terms BED and eHealth were used to develop the algorithms. Examples of terms include bing*, *binge eating*, *web-based treatment*, *internet*, *mobile phone*, *smartphone*, *telemedicine*, *telehealth*, and *remote*. Searches were performed until February 2019 and returned a total of 744 results. Articles were restricted to English and randomized controlled trials (RCTs), where possible. In addition, 2 articles were found via *FasebJ* and ResearchGate. A detailed outline of the literature search is presented in Figure 1.

### Inclusion Criteria

Studies used in the analysis had to meet the following prespecified inclusion criteria: (1) adult male or female participants aged 18 years and older, diagnosed with full or subthreshold BED. In the latter case, patients had to meet the criterion for objective binge episodes (OBEs) but could lack one of the other Diagnostic and Statistical Manual of Mental Disorders criteria (ie, frequency of less than 2 days with OBEs in 6 months, no marked distress, or presence of only 2 of the 5 associated criteria) [1,33,37] (both full and subthreshold BED participants were included, as a recent study [38] showed that individuals with these conditions do not differ significantly on measures of weight and shape concerns, restraint, psychiatric distress, and history of seeking treatment for an eating or weight problem [38]); (2) the intervention was a form of eHealth treatment and was the main form of treatment and was not administered posttreatment as a form of outpatient care; (3) outcomes observed included at least one of the following: OBE, BMI, Eating Disorder Examination Questionnaire (EDE-Q) total score, EDE-Q weight concern, and EDE-Q shape concern. The EDE-Q is a self-report questionnaire that measures ED severity and consists of 4 subscales: restraint, eating concern, weight concern, and shape concern [39]. This study explores total scores and relevant subscales that are related to the cognitive-affective aspect of body image (ie, shape and weight concern), as body image disturbances are common in this population [40]; (4) included studies must be RCTs, in which participants were randomly allocated to either a treatment or control group. This was to ensure that a robust methodology was used in each study and ensure that all estimates of effectiveness were not confounded by other factors and to enable the valid pooling of the data; (5) the comparison or control group consisted of a waitlist (WL) or no treatment; and (6) mean and SD pre- and posttreatment (or data available to calculate them) were reported. Studies that analyzed BED with other disorders were excluded if they did not stratify the ED of interest. If the eHealth component was outpatient, used subsequent to another main treatment, and not the main form of treatment, the study was excluded.

### Data Extraction and Risk of Bias Assessment

The following data were extracted from each study: (1) first author’s last name, (2) year of publication, (3) total sample size and group size posttreatment, (4) type of treatment and control, (5) BED diagnosis status and criteria used for diagnosis, (6) mean age of participants, (7) treatment length, (8) therapist contact, (9) percentage of females in the study, and (10) mean and SD posttreatment for all outcomes in the treatment and control groups.

The overall risk of bias was assessed by 2 independent reviewers using the revised Cochrane risk of bias tool for randomized trials [41]. Any disagreements in the assessments were resolved via discussion. The following 5 domains were assessed: (1) bias arising from the randomization process, (2) bias due to deviations from intended interventions, (3) bias due to missing outcome data, (4) bias in outcome measurement, and (5) bias in selection of the reported result. Each study was summarized as having a low, medium, or high risk of bias (Tables 1 and 2).

**Table 1:** Characteristics of the studies included in the meta-analysis. Waitlist controls were compared with an internet version of cognitive behavioral therapy or guided self-help therapy. All 3 therapies use the principles of cognitive behavioral therapy and have varying degrees of therapist guidance and interaction with the patients.

Authors	Participant				BED^a^ diagnosis (diagnostic criteria)		Therapist contact		Females, n (%)		Age, mean (SD)		Treatment length		Risk of bias
	Total, N	Treatment group, n (%)	Control group, n (%)												
Carrard et al, 2011A [31]	74	37 (50) I-GSH^b^	37 (50) WL^c^	Full (n=43) or subthreshold (n=31) BED (eating disorder questionnaire based on DSM-IV^d^)		Weekly contact via email		74 (100)		36 (11.4)		6 months		Low	
ter Huurne et al, 2015 [30]	85	43 (51) I-CBT^e^	42 (49) WL	Diagnosed with BED (participant self-report based on DSM-IV)		Internet-based contact with the therapist twice a week		85 (100)		40.2 (11.4)		15 weeks		Low	
Wagner et al, 2016 [10]	139	69 (50) I-CBT	70 (50) WL	Diagnosed with BED (telephone interview using DSM-5^f^ criteria)		Internet-based contact with therapist when submitting assignments		134 (96.4)		35.1 (9.9)		16 weeks		Low	

^a^BED: binge eating disorder.

^b^I-GSH: internet-guided self-help.

^c^WL: waitlist.

^d^DSM-IV: Diagnostic and Statistical Manual of Mental Disorders, 4th edition.

^e^I-CBT: internet-based cognitive behavioral therapy.

^f^DSM-5: Diagnostic and Statistical Manual of Mental Disorders, 5th edition.

**Table 2:** Postreatment results for each study.

Authors	OBE^a^			BMI			EDE-Q^b^			Shape concern			Weight concern	
	Treatment posttreatment, mean (SD)	Control posttreatment, mean (SD)	Treatment posttreatment, mean (SD)		Control posttreatment, mean (SD)	Treatment posttreatment, mean (SD)		Control posttreatment, mean (SD)	Treatment posttreatment, mean (SD)		Control posttreatment, mean (SD)	Treatment posttreatment, mean (SD)		Control posttreatment, mean (SD)
Carrard et al, 2011A [31]	5.5 (7.4)	9.1 (8.8)	29.2 (6.0)		27.9 (5.4)	2.5 (1.1)		2.9 (1.0)	3.7 (1.3)		4.1 (1.3)	—^c^		—
ter Huurne et al, 2015 [30]	—	—	—		—	2.6 (1.3)		3.2 (0.9)	3.5 (1.6)		4.2 (1.1)	3.1 (1.4)		3.9 (0.9)
Wagner et al, 2016 [10]	6.8 (7.5)	14.9 (7.7)	31.4 (6.9)		32.8 (8.3)	2.5 (1.2)		3.7 (0.8)	3.4 (1.4)		4.5 (0.8)	3.0 (1.3)		4.2 (0.8)

^a^OBE: objective binge episode.

^b^EDE-Q: Eating Disorder Examination Questionnaire.

^c^Not available (missing data).

### Statistical Analysis

Given the anticipated heterogeneity, the studies were pooled using a meta-analytic random effects model. The reported *P* values were two-sided, with *P*<.05 considered statistically significant. As all outcomes measured were continuous, means and SD were used to calculate effect size, which was expressed as a standardized mean difference (SMD), corrected using Hedges *g* for a small sample size to reduce positive bias. The SMD was used to ensure that the scales used in the different studies were standardized. SMDs were classified based on the level of effect: less than 0.20 signified a very small effect, 0.20 signified a small effect, 0.50 signified a medium effect, and 0.80 signified a large effect. All studies used in the analysis reported mean and SD values. The I^2^ statistic [42] was used to quantify between-study heterogeneity. Here, the percentage of total variation in the estimates of the effect due to between-study heterogeneity is reported [43]. I^2^ values above 25% indicated low heterogeneity, 50% indicated moderate heterogeneity, and above 75% indicated substantial heterogeneity [43]. The restricted maximum likelihood model estimator was used to measure the between-study variance, τ^2^ [44]. Data were analyzed using the statistical software package RStudio Desktop version 1.1.463, metafor package [45].

## Results

### Study Characteristics

In total, 3 RCT studies met all the inclusion criteria and were included in the meta-analysis, with a total of 298 participants. All studies were consistent in the intervention and study participants and were appropriately combined in a meta-analysis. Assessment of the risk of bias scores indicated a low risk of bias in all 3 studies. Furthermore, all the studies recruited female participants, except 1 that sampled 96.4% (134/139) females [10]. The mean age of participants ranged from 35.1 (SD 9.9) to 40.2 (SD 11.4) years. In 2 studies, all participants were diagnosed with full BED. In one study [31], participants consisted of a combination of full (n=43) or subthreshold (n=31) BED. Furthermore, the method of BED diagnosis varied among the studies; in 2 studies, diagnosis was made using the Diagnostic and Statistical Manual of Mental Disorders, 4th edition (DSM-IV) criteria, and in 1 study, the Diagnostic and Statistical Manual of Mental Disorders, 5th edition (DSM-5) criteria were used. Among the studies, 2 administered I-CBT as the treatment, and the other study administered an I-GSH, which also used CBT principles. Although all 3 studies were based on CBT principles, they had varying degrees of therapist contact, which ranged from weekly to any time the participant submitted an assignment. None of the studies were unguided. All the therapists in the study were clinical psychologists, except one [30] where therapists either had a Bachelor of Science in nursing or social work or a Master of Science in psychology. The studies also varied in treatment length, ranging from 15 weeks to approximately 26 weeks (6 months). One of the studies did not measure OBE or BMI [30], and another study did not report EDE-Q weight concern [31]. The characteristics of the included studies are summarized in Tables 1 and 2.

### Quantitative Analysis

#### Overview

A summary of the meta-analysis results for each included outcome is described below and summarized in Table 3. Forest plots for each outcome are presented in Multimedia Appendix 2 [10,30,31].

**Table 3:** Summary of findings for the randomized controlled trial studies.

Outcome	Studies, n (%)	Participants, N	Effect size; SMD^a^ (95% CI)	Heterogeneity, I^2b^ (%)	*P* value
Objective binge episodes^c^	2 (67)	213	−0.77 (−1.38 to −0.16)	77	.01
BMI	2 (67)	213	−0.01 (−0.40 to 0.39)	50	.96
EDE-Q^d^ total^c^	3 (100)	298	−0.71 (−1.20 to −0.22)	77	.005
Shape concern^c^	2 (67)	298	−0.61 (−1.01 to −0.22)	64	.002
Weight concern^c^	2 (67)	224	−0.91 (−1.33 to −0.48)	56	<.001

^a^SMD: standardized mean difference.

^b^I^2^ values above 25% indicated low heterogeneity, 50% indicated moderate heterogeneity, and above 75% indicated substantial heterogeneity [43].

^c^*P*<.05.

^d^EDE-Q: Eating Disorder Examination Questionnaire.

#### OBE Result

In total, 2 of the studies involving 213 participants evaluated OBE. Among the studies, one demonstrated a significant reduction in OBE in the treatment group compared with the WL control (SMD −1.06, 95% CI −1.42 to −0.70). The pooled SMD was −0.77 (95% CI −1.38 to −0.16; Figure S1 of Multimedia Appendix 2 and Table 3), which showed a statistically significant effect.

#### BMI Result

In total, 2 of the studies involving 213 participants evaluated BMI. None of the studies demonstrated a significant change in BMI in the treatment group compared with the WL group. The pooled SMD was −0.01 (95% CI −0.40 to −0.39; Figure S2 of Multimedia Appendix 2 and Table 3).

#### EDE-Q Total Score

In total, 3 of the studies involving 298 participants evaluated the EDE-Q total score. Two of the studies demonstrated a significant reduction in EDE-Q scores in the treatment group compared with WL groups (SMD −1.17, 95% CI −1.53 to −0.81 and SMD −0.53, 95% CI −0.96 to −0.10). The pooled SMD was statistically significant with an estimate of −0.71 (95% CI −1.20 to −0.22; Figure S3 of Multimedia Appendix 2 and Table 3).

#### EDE-Q Shape Concern

In total, 3 of the studies involving 298 participants evaluated the EDE-Q subscale of shape concern. Two of the studies demonstrated a significant reduction in shape concern scores in the treatment group compared with WL groups (SMD −0.96, 95% CI −1.31 to −0.61 and SMD −0.50, 95% CI −0.94 to −0.07). The pooled SMD was −0.61 (95% CI −1.01 to −0.22; Figure S4 of Multimedia Appendix 2 and Table 3).

#### EDE-Q Weight Concern

In total, 2 of the studies involving 224 participants evaluated the EDE-Q subscale of weight concern. Both studies demonstrated a significant reduction in shape concern scores in the treatment group compared with WL groups (SMD −1.11, 95% CI −1.47 to −0.75 and SMD −0.67, 95% CI −1.11 to −0.23). The pooled SMD is −0.91 (95% CI −1.33 to −0.48; Figure S5 of Multimedia Appendix 2 and Table 3).

## Discussion

### Principal Findings

This study reports the first meta-analysis of RCTs designed to assess the efficacy of eHealth treatments for individuals diagnosed with BED. Due to its specificity, 3 studies met the inclusion criteria and were included in the analysis. All of these used an internet-based form of guided CBT therapy, wherein the degree of therapist interaction varied depending on the nature of the intervention that was administered. Due to the novelty of eHealth innovations and our study objectives, it was important to evaluate efficacy by restricting to the RCT design. Despite the limited number of studies, statistically significant results demonstrated the effectiveness of internet-based CBT, in combination with GSH treatment, in reducing binge episodes, ED psychopathology, and shape and weight concerns. Although the efficacy of conventional CBT therapy has been well demonstrated [46-48], currently there is insufficient data to claim that internet-based CBT has an evidence base or effect size that is comparable with in-person CBT.

Despite the moderate effect of the treatment in reducing the number of OBEs, internet-based therapies did not appear to produce a significant change in BMI. Notably, the lack of substantial weight loss has long been considered one of the principal drawbacks of the current CBT therapy for BED. For instance, Peat et al [49] found no significant difference in BMI between therapist-led, partially therapist-led, and structured self-help CBT. Similarly, in a recent meta-analysis comparing pharmacological, psychological, and combined treatments, CBT was effective in reducing binge episodes, but its effect on weight loss was minimal [50]. Furthermore, the impact on BMI may be indirect, via reduced binge frequency, and consists of an extended maintenance phase [51]. In a more recent study involving in-person CBT treatment, no significant change in BMI was observed between pre- and posttreatment assessments [52]. These results are also in line with a study comparing in-person and eHealth CBT treatments, where BMI did not decrease in either treatment group, nor were they significantly different between the two groups [33]. Taken together, these results indicate that regardless of how CBT is administered, its effects on weight loss are minimal to nonexistent.

In patients with BED, the purpose of CBT is to reduce binge eating frequency and body image dissatisfaction by altering destructive behaviors and thinking patterns, particularly those that involve eating, weight and shape, and psychosocial functioning [53]. This is in line with the findings of the current meta-analysis, where a moderate effect of the treatment in reducing shape and weight concerns was observed. There is no direct treatment emphasis on diet or weight loss. Indeed, it has been observed that eating patterns may shift from binging to less compulsive overeating, which does not have the same elements of guilt and compulsion associated with it. In this way, the caloric deficits may not be met, and consequently, BMI does not change. However, research has also found that a complete abstinence from binges is associated with significant improvements in dietary and psychological outcomes, which may improve weight status in the long term [54]. Although there was a significant reduction in binge episodes in this meta-analysis, none of the treatment studies included in this analysis resulted in complete abstinence following treatment. Interestingly, a recent study has shown that when compared with obese, non-BED participants, those diagnosed with BED have a significantly higher threshold for what comprises a *large amount of food* [55]. Furthermore, laboratory-based studies have also demonstrated that when compared with their non-BED counterparts, participants diagnosed with BED tend to have a significantly higher caloric intake and consume large amounts of food even during nonbinge-eating episodes [56,57].

In addition to exploring the efficacy of eHealth interventions, this meta-analysis highlights an important point regarding the status of eHealth and BED research. That is, despite its promising impact on improving BED symptomatology and its moderate effect in reducing ED psychopathology, there are only a small number of RCT studies that have evaluated the efficacy of eHealth treatments, and even fewer studies have compared them with the traditional, in-person method of delivery [33,34]. Specifically, only 2 other studies compared eHealth treatments with in-person therapy [33,34]. Although the efficacy of internet treatments was demonstrated in both studies, when compared with in-person treatment, one study reported inferiority [33] and the other found no significant differences between the 2 treatment types [34]. Although the results of these studies are promising, further research will provide a better understanding of the merits of internet versus in-person treatment. However, our work shows that internet treatment is efficacious and may represent a more accessible and cost-effective treatment option for those who have difficulty obtaining in-person treatment for their ED. Given that BED is the most common ED and that most individuals diagnosed do not seek treatment, it is vital for more accessible modes of treatment delivery to be assessed for their efficacy.

Despite improving the accessibility of treatment delivery, an important point that may warrant further analysis is *who* is administering the treatment. In this analysis, the smallest treatment effect was demonstrated in the study by ter Huurne et al [30]. Importantly, this was the only study that did not have clinical psychologists guiding the treatment. It may be that the specialized experience of a clinical psychologist has a stronger impact on the treatment compared with less experienced medical health professionals. However, research has determined that the efficacy of treatment, including CBT, for other mental health disorders is similar between clinical psychologists and appropriately trained medical health professionals [58-61]. It is important for future studies to determine whether these results can be generalized to patients with BED.

Since January 2019, 3 RCT study protocols have been published that outline the use of eHealth interventions for the treatment of BED [15,62,63]. All 3 propose using an internet-based form of CBT treatment and will use the DSM-5 criteria to diagnose BED. This is in contrast to this meta-analysis, in which of the 3 published studies, 2 used the DSM-IV criteria. One of the added benefits of using the DSM-5 criteria is that it may also increase participant recruitment for these studies. It has already been demonstrated that many health care providers and psychiatrists have difficulty identifying BED symptoms, leading to a greater need to improve the knowledge of the diagnostic criteria for BED [64]. With the inclusion of BED as a stand-alone diagnosis and the resulting increase in awareness of the disorder, participant recruitment for these studies may increase, providing a more accurate depiction of the efficacy of these eHealth treatments. This was also a phenomenon observed in the meta-analysis; when observing the effects of individual studies, the study with the largest sample size [10] was also the one that used the DSM-5 criteria. As a result, this study also found the largest treatment effect and narrowest CIs for all outcomes when compared with the other studies used in the analysis.

### Limitations

One of the limitations of this study is its level of generalizability. The majority of participants were middle-aged women who were overweight or obese. Therefore, how well these findings can be applied to other age groups and male patients is not clear. It is important to note, however, that one of the reasons why the participant pool consists primarily of overweight and obese women may be that the disorder has a higher prevalence in females, and those diagnosed are 3-6 times more likely to be obese [65]. Furthermore, the higher prevalence of the disorder in women may enhance the efficacy and use of these eHealth treatments, as women are more likely to use the internet for medical and health-related information [66]. Another limitation of the study was that despite similarities in the study designs, heterogeneity was still quite high. The heterogeneity of the outcomes BMI, shape, and weight concern were in the moderate range; however, for the outcomes OBE and EDE-Q total, their values indicated substantial heterogeneity (77% for both). There are several reasons why this might have occurred. First, there was considerable variability in the criteria used to diagnose BED among the studies included in the analysis. In total, 2 of the studies used the DSM-IV criteria (1 relying on participant self-report) and 1 used the DSM-5 criteria. Compared with the DSM-IV, the use of the DSM-5 yields higher prevalence rates of BED and more accurate criteria for diagnosis [67-69]. In the analysis, the only study using the DSM-5 criteria not only had a higher sample size but also the largest effect size. Second, although the CBT-I and GSH-I treatments in the study were all based on CBT principles, the treatment protocols and duration were quite different. Finally, because of the limited number of studies and the limited participant pool, the results must be interpreted with caution.

### Conclusions

This study provides preliminary evidence that eHealth treatments, and more specifically internet-based guided CBT treatments, are appropriate treatment avenues for BED. However, because of the limited number of published RCTs in this field, it is important for the current evidence base to become more complete, so that more conclusive results can be extracted. As more findings are published in this area, future studies not only need to analyze the efficacy of eHealth treatments but to further hone in on the effectiveness of in-person versus eHealth treatments, to investigate the differences in efficacy among the different types of eHealth treatments, to evaluate which elements of the treatment result in unchanged BMI, and to determine the characteristics of patients with BED that make them more suitable candidates for this alternative form of treatment.

## Appendix

Multimedia Appendix 1 PRISMA (Preferred Reporting Items for Systematic Reviews and Meta-Analyses) checklist.

Multimedia Appendix 2 Forest plot of outcomes.

## Abbreviations

BED: binge eating disorder
CBT: cognitive behavioral therapy
DSM-IV: Diagnostic and Statistical Manual of Mental Disorders, 4th edition
DSM-5: Diagnostic and Statistical Manual of Mental Disorders, 5th edition
ED: eating disorder
EDE-Q: Eating Disorder Examination Questionnaire
I-CBT: internet-based cognitive behavioral therapy
I-GSH: internet-based guided self-help
MeSH: Medical Subject Headings
OBE: objective binge episode
PRISMA: Preferred Reporting Items for Systematic Reviews and Meta-analyses
RCT: randomized controlled trial
SMD: standardized mean difference
WL: waitlist
