# Supplementary material for: The Efficacy of eHealth Interventions for the Treatment of Adults Diagnosed With Full or Subthreshold Binge Eating Disorder: Systematic Review and Meta-analysis
Source: J Med Internet Res. 2021 Jul 20;23(7):e17874. doi: 10.2196/17874 (PMC8335602; doi:10.2196/17874)
Supplement: Multimedia Appendix 2 [file jmir_v23i7e17874_app2.docx]

**Forest plots of outcomes**


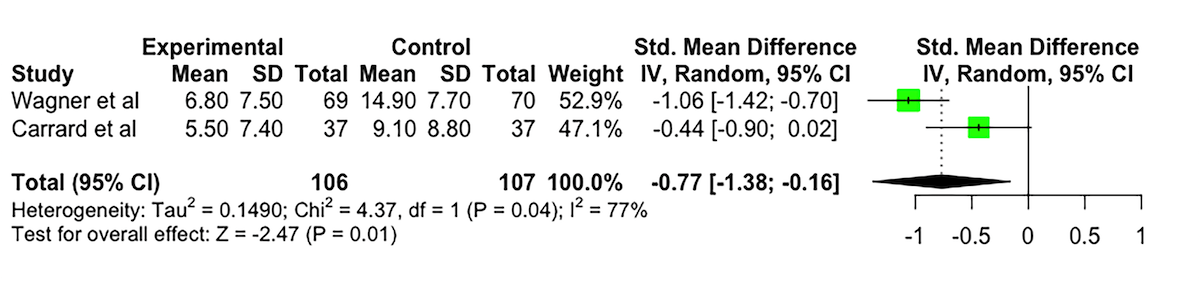


Figure S1. Forest plot of the SMDs with corresponding 95% CIs of studies corresponding to the effect of internet treatment compared to WL control on OBE.


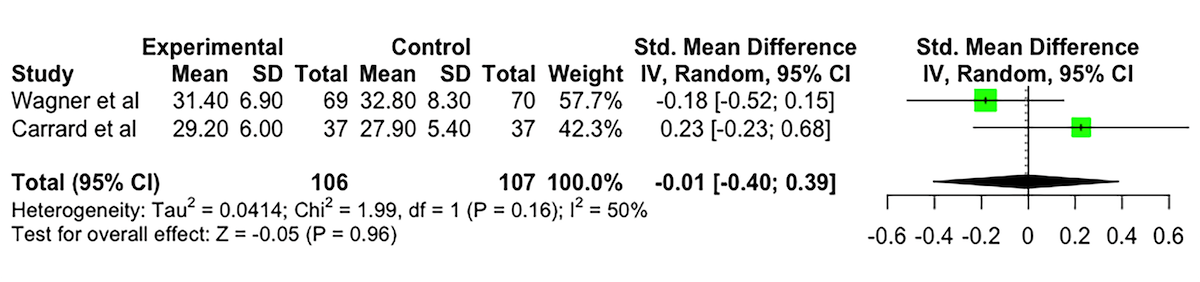


Figure S2. Forest plot of the SMDs with corresponding 95% CIs of studies corresponding to the effect of internet treatment compared to WL control on BMI.


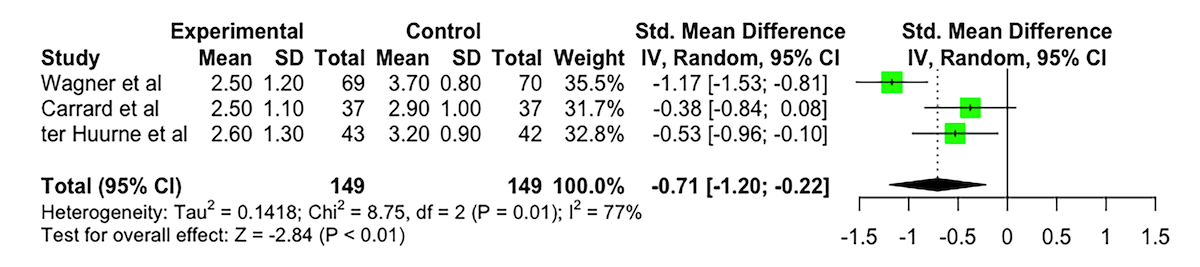


Figure S3. Forest plot of the SMDs with corresponding 95% CIs of studies corresponding to the effect of internet treatment compared to WL control on EDE-Q total score.


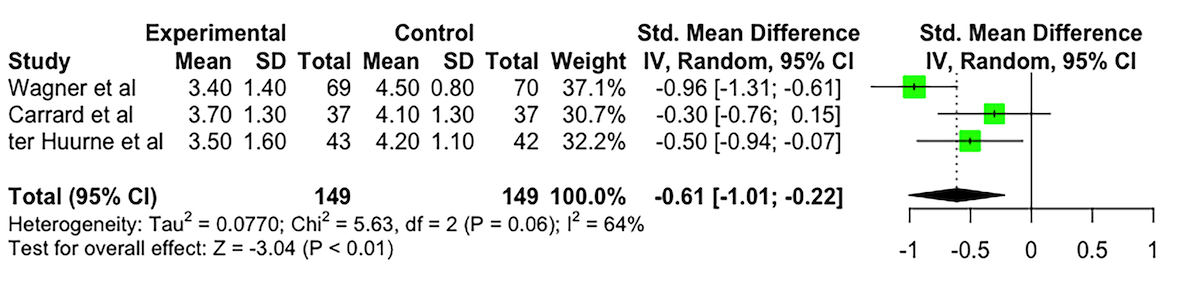


Figure S4. Forest plot of the SMDs with corresponding 95% CIs of studies corresponding to the effect of internet treatment compared to WL control on the EDE-Q subscale, shape concern.


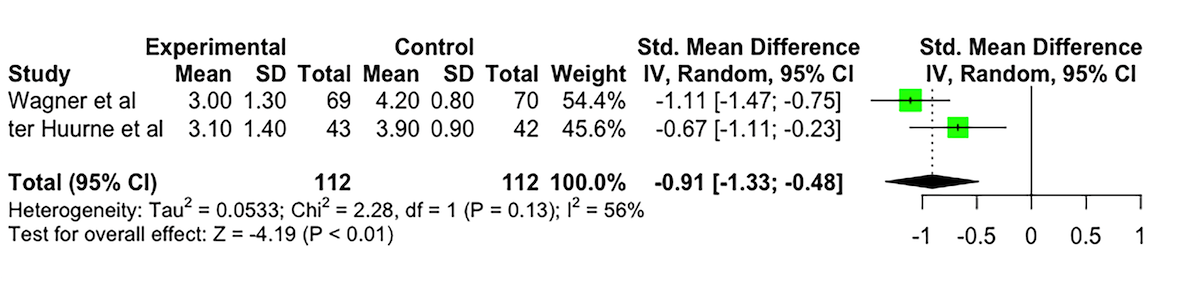
Figure S5. Forest plot of the SMDs with corresponding 95% CIs of studies corresponding to the effect of internet treatment compared to WL control on the EDE-Q subscale, weight concern.

This is a Multimedia Appendix to a full manuscript published in the J Med Internet Res. For full copyright and citation information see http://dx.doi.org/10.2196/jmir.17874
